# Brain Distribution and Metabolism of Flupirtine, a Nonopioid Analgesic Drug with Antiseizure Effects, in Neonatal Rats

**DOI:** 10.3390/pharmaceutics10040281

**Published:** 2018-12-16

**Authors:** Madhoosudan A. Patil, Brock A. Matter, Yogendra H. Raol, David W. A. Bourne, Ryan A. Kelley, Uday B. Kompella

**Affiliations:** 1 Department of Pharmaceutical Sciences, University of Colorado Anschutz Medical Campus, Aurora, CO 80045, USA; madhoosudan.patil@ucdenver.edu (M.A.P.); brock.matter@ucdenver.edu (B.A.M.); david.bourne@ucdenver.edu (D.W.A.B.); ryan.a.kelley@ucdenver.edu (R.A.K.); 2 Department of Pediatrics, Division of Neurology, School of Medicine, University of Colorado, Anschutz Medical Campus, Aurora, CO 80045, USA; yogendra.raol@ucdenver.edu; 3 Department of Ophthalmology, University of Colorado Anschutz Medical Campus, Aurora, CO 80045, USA; 4 Department of Bioengineering, University of Colorado Anschutz Medical Campus, Aurora, CO 80045, USA; 5 Colorado Center for Nanomedicine and Nanosafety, University of Colorado Anschutz Medical Campus, Aurora, CO 80045, USA

**Keywords:** flupirtine, D-13223, brain delivery, drug metabolism

## Abstract

Flupirtine, a nonopioid analgesic drug, is effective in treating neonatal seizures. However, its brain delivery and pharmacokinetics are unknown in neonatal mammals. The purpose of this study was to determine the pharmacokinetics of flupirtine and the formation of its active metabolite D-13223 in various tissues such as brain in neonate animals. On postnatal day 7, rat pups received 25 mg/kg of flupirtine intraperitoneally. Liver; heart; kidney; lung; spleen; retina; serum; and brain regions hippocampus, cortex, and the remaining brain (devoid of cerebellum) were harvested up to 24-h postdosing. An LC-MS/MS assay was developed to quantify flupirtine and D-13223. Flupirtine was delivered to all tissues assessed, with the highest area under the concentration vs. time curve (AUC_0–24h_) in liver (488 µg·h/g tissue) and the lowest in spleen (82 µg·h/g tissue). Flupirtine reached the brain, including the hippocampus and cortex, within 1 h of dosing and persisted at 24 h. Flupirtine AUC in various brain regions was approximately 195 µg·h/g tissue. The half-life of flupirtine in various tissues ranged from 3.1 to 5.2 h. D-13223 was formed in vivo and detected in all tissues assessed, with the concentrations being the highest in the liver. Incubation of isolated neonatal rat liver, heart, kidney, lung, spleen, whole eye, serum, or whole brain with flupirtine for 3 h at 37 °C formed D-13223 in all tissues, except serum. D-13223 formation was the highest in isolated liver tissue. Tissue partition coefficients based on isolated tissue uptake correlated well with in vivo tissue:serum drug exposure ratios. Thus, flupirtine reaches the target brain tissues from the systemic route in neonatal rats, and brain tissue forms the active metabolite D-13223.

## 1. Introduction

Flupirtine (ethyl-*N*-[2-amino-6-(4-fluoro-phenylmethylamino) pyridin-3-yl] carbamate), an aminopyridine, is a centrally acting nonopiate analgesic that was first approved in Europe in 1984 and is now marketed in India, China, and Brazil for treating acute and chronic pain in patients. It is effective against pain involving the musculoskeletal system, tension headache, tumor pain, pain associated with dysmenorrhea, and pain following traumatic/orthopedic surgery and injuries [1,2,3]. Flupirtine acts primarily by opening KCNQ-type potassium (K^+^) channels during the early depolarization phase, thereby increasing the threshold for generating a neuronal action potential [4,5]. It activates G-protein-regulated, inwardly rectifying K^+^ channels (GIRKs) and stabilizes the membrane resting potential by indirectly inhibiting the NMDA receptor activity [4,6,7,8,9,10]. Additionally, flupirtine shifts the gating of γ-aminobutyric acid (GABA) type-A receptors to a lower GABA concentration [4,5,10]. 

Besides being an effective analgesic, studies in animal models suggest that flupirtine has therapeutic potential in treating neurological disorders. Flupirtine reduced brain injury, promoted remodeling of the brain tissue, and reduced cognitive deficits in animal models of ischemia in adults [11,12,13]. More recent studies suggest that flupirtine is a highly effective treatment for neonatal seizures in global hypoxia, hypoxic–ischemic encephalopathy (HIE), flurothyl, and kainic acid animal models and is more efficacious than the current first-line anticonvulsant drugs [14,15,16]. The effectiveness of flupirtine against neonatal seizures in multiple animal models provides strong evidence for assessing this medication in newborn humans with seizures. However, there is insufficient literature concerning the metabolism and distribution of flupirtine in various tissues after systemic administration in neonates. 

Flupirtine is well absorbed from the gastrointestinal tract in humans with a bioavailability of 90% via the oral route and 70% via the rectal route [17]. The plasma half-life of flupirtine after oral or intravenous administration in adult humans ranges from 6.5 to 9.5 h [18,19]. The half-life values are similar in epileptic patients [20]. The mean elimination half-life of flupirtine is longer in elderly than in younger subjects, which could be due to decreased renal clearance and hepatic metabolism in older patients [18]. Following oral administration, the plasma half-life of flupirtine in adult rats, dogs, and cats is 2.2, 2.6 [21], and 13.6 h [22], respectively. In adult horses, the plasma half-life of flupirtine is 3.02 h after intravenous administration [23]. However, similar information is not available for flupirtine in neonatal animals. Furthermore, biodistribution of flupirtine to brain and tissues other than blood/plasma was not previously reported in any animal model. Determining biodistribution, metabolism, and pharmacokinetics of flupirtine will support its potential use in a variety of neonatal diseases. 

In the current study, we determined the tissue distribution, elimination rate constant, and half-life of flupirtine in neonatal rats after its systemic administration. Since D-13223 is an active metabolite of flupirtine [21], we also determined its concentrations in various tissues. Because it was not possible to dissect the contribution of various tissues to flupirtine metabolism observed in vivo, due to rapid blood exchange between tissues, we quantified the metabolism of flupirtine by freshly isolated tissues as well. Finally, we determined whether the ex vivo (isolated tissue incubation) tissue partition coefficient correlated with the in vivo tissue:serum drug exposure ratio.

## 2. Materials and Methods

### 2.1. Materials

Dimethyl sulfoxide (DMSO), flupirtine maleate, flupirtine-d_4_ maleate, and formic acid were obtained from Sigma-Aldrich and were of UltraPure quality (Sigma-Aldrich Chemicals company, St. Louis, MO, USA). Optima LC-MS grade acetonitrile and HPLC grade methyl tert-butyl ether (MTBE) were purchased from Fisher Scientific (Fisher Scientific, Hampton, NH, USA). D-13223 HCl was obtained from Santa Cruz Biotechnology (Santa Cruz Biotechnology, Dallas, TX, USA).

### 2.2. Methods

#### 2.2.1. Animals

All procedures involving animals were performed in accordance with the NIH guidelines for the care and use of laboratory animals and according to the protocol approved by the Institutional Animal Care and Use Committee (IACUC), University of Colorado Anschutz Medical Campus (UC-AMC) (Protocol number—B-98104(12)1E; date of approval—Oct 22, 2015). Additionally, efforts were made to reduce animal suffering and the number of animals used. Timed pregnant Sprague-Dawley rats were obtained from Charles River Laboratories (Wilmington, MA). The pregnant rats were at the 14th day of gestation (E14) on arrival at the vivarium and delivered the pups at E22 or E23. Pups from both sexes were used for the study. 

#### 2.2.2. Tissue Distribution Studies

For in vivo studies, flupirtine maleate was dissolved in DMSO at a concentration of 12 mg/mL and injected intraperitoneally at the dose of 25 mg/kg body weight to P7 rat pups (male and female). Animals were anesthetized at 1, 6, and 24 h using isoflurane, and ~200 µL of blood was drawn from cardiac puncture, allowed to clot, and centrifuged to isolate the serum. Each animal was euthanized by transcardial perfusion with 10 mL of sterile ice-cold PBS at pH 7.4. Right cortex, left cortex, right hippocampus, left hippocampus, remaining brain excluding cerebellum (remaining brain), heart, kidney, liver, lung, right retina, left retina, and spleen were isolated from each animal and weighed. All the surgical tools used were either replaced or cleaned by rinsing in 1N nitric acid, 1 L double-distilled water, followed by DMSO and 1 L double-distilled water, and then blotted dry between each animal to prevent any transfer of drug between tissue samples. Dissected tissues were immediately frozen on dry ice and stored at −80 °C pending analysis. Typical tissue preparation and LC-MS/MS analysis are further described below.

#### 2.2.3. Sample Preparation for In Vivo Tissue Distribution 

Tissues (liver, spleen, kidney, heart, and lung) were mixed with 10 µL of water per milligram of tissue and homogenized in a glass–glass homogenizer. Fibrous tissues were chopped with a fresh razor blade prior to homogenization. All tools used were cleaned between samples. Homogenized samples were stored at −20 °C until extracted. An aliquot containing ~30 mg of homogenized tissue was taken from each sample for extraction. Homogenates were spiked with flupirtine-d_4_ (internal standard) and diluted with water.

Cortex, hippocampus, and remaining brain tissue (all about 30 mg); retina samples (entire retina); and serum samples (30 µL aliquots) were directly added to microcentrifuge tubes. Samples were diluted with water, spiked with flupirtine-d_4_, and homogenized (serum samples did not require homogenization) by shearing with a pipette followed by sonication for 5 min.

Samples were mixed with 60 µL of 0.1N NaOH, vortexed, bath sonicated for 5 min, and mixed with 1 mL of MTBE. Sample solutions were vortexed and bath sonicated for 5 min, then spun down at 10,000 rcf for 5 min. The supernatant was collected into fresh microcentrifuge tubes capped with hole-punched lids and dried in a Savant™ Speedvac™, Model SC100. Dried samples were stored at −20 °C until analysis. Immediately prior to LC-MS/MS analysis, dry samples were reconstituted in water and loaded into 96-well microtiter plates. 

#### 2.2.4. Flupirtine Metabolism in Isolated Tissues

Another set of P7–P9 SD rat pups (male and female) were anesthetized with isoflurane and ~200 µL of blood was drawn from cardiac puncture, allowed to clot, and centrifuged to isolate the serum. Animals were euthanized by transcardial perfusion with 10 mL of prewarmed (37 °C) PBS. Whole brain, heart, kidney, liver, lung, spleen, and eyes were isolated. Immediately after isolation, a piece of each tissue type weighing 100 (±10) mg was isolated with a blade and placed on a microscope slide and cut into four approximately equal pieces. As the total weight of both the eyes was less than 100 mg, the two eyes were combined (total weight ~77 mg) after each eye was cut into two pieces and incubated together to represent one sample. Tissue pieces were then suspended in glass scintillation vials with 994 µL of prewarmed (37 °C) sterile PBS (pH 7.4). Flupirtine maleate (60 µg in 6 µL) from a freshly prepared stock in DMSO (10 mg/mL) solution was added to all the tissues and incubated at 37 °C with rocking (150 rpm) in a water bath incubator. At 0.25, 0.5, 1, 2 and 3 h, 100-µL aliquots of the tissue incubation medium were removed and placed in separate ice-cold plastic vials, which were stored at −80 °C until analysis. Lost sample volume (100 µL) was replenished with fresh sterile prewarmed (37 °C) PBS to maintain the volume of 1 mL and the incubation was continued at 37 °C with rocking.

#### 2.2.5. Sample Preparation for Ex Vivo Metabolism

Collected aliquots from tissue samples were spun down to remove any tissue debris and 5 µL of the supernatant was diluted into 2 mL of water containing 400 pmol of flupirtine-d_4_ and analyzed by LC-MS/MS. Each serum sample aliquot (5 µL) was diluted into 300 µL of water containing 400 pmol of flupirtine-d_4_ and then extracted with MTBE as previously described for in vivo samples. Serum sample extracts were reconstituted in 1 mL of water and analyzed by LC-MS/MS.

#### 2.2.6. LC-MS/MS Analysis

A Prominence HPLC (Shimadzu, Kyoto, Japan) coupled to a QTrap 4500 mass spectrometer (AB Sciex, Framingham, MA, USA) operated in ESI^+^ mode was used for the analysis. Optimized parameters were found to be: curtain gas, 45 psi; collision gas, high; ion spray voltage, 3000 V; source temperature, 650 °C; ion source gas 1 and 2, 60 psi; declustering potential, 50 V; and collision energy, 24 V.

Analytical separation and quantitation of flupirtine (*m/z* 305.1 → *m/z* 196.1), flupirtine-d_4_ (*m/z* 309.1 → *m/z* 196.1), and D-13223 (*m/z* 275.2 → *m/z* 166.1) were achieved on an Agilent Eclipse PLUS-C18 column (4.6 × 150 mm, 5 µm) (Agilent Technologies, Santa Clara, CA, USA). The column was held at 40 °C and eluted at 1 mL/min with a gradient of 0.1% formic acid in water (A) and 0.1% formic acid in acetonitrile (B). Chromatographic separation was achieved with a linear gradient (time, % of solvent B): 0–12 min, 10%–30% B; 12–13 min, 30%–10% B; then isocratic for 9 min at 10% B to re-equilibrate the column. Limited validation of the method was performed. The lower limit of quantitation (LLOQ) for flupirtine using flupirtine-d_4_ internal standard was at least 5 fmol (accuracy = 100.5%, relative standard deviation (RSD) = 1.3%; 0.42 ng/mL of flupirtine maleate; N = 4) and for D-13223 using the flupirtine-d_4_ internal standard, the LLOQ was 0.5 fmol (accuracy = 98.4%, RSD = 2.2%, 0.031 ng/mL of D-13223 HCl; N = 4). 

The concentration of flupirtine was determined by comparing the ratio of the HPLC-ESI^+^-MS/MS peak area for flupirtine to the flupirtine-d_4_ peak area against the area ratios generated from known concentrations of flupirtine and flupirtine-d_4_ in a calibration curve. 

The MS/MS response for flupirtine was linear from <125 fmol to ~7.5 pmol on column (0.011–0.63 µg/mL flupirtine maleate). The internal standard flupirtine-d_4_ was typically used at 2.5 pmol on the column (0.21 µg/mL flupirtine-d_4_ maleate). Flupirtine calibration curves were similar in both water and liver tissue homogenates; therefore, calibration curves in water were typically used. Further, the matrix effect on flupirtine and D-13223 analysis was assessed using a dynamic method [24] based on postcolumn infusions and liver tissue as a representative matrix. Extracts from homogenized liver were analyzed by LC-MS/MS; then, the analysis was repeated with a 25 nM (10.51 ng/mL) of flupirtine maleate solution and 25 nM (7.77 ng/mL) of D-13223 HCl solution infused at 10 µL/minute into the column eluent through a three-way junction. Tissue matrix did not show any interfering peaks. Further, no matrix-based suppression or enhancement of the flupirtine or the D-13223 signals were observed (see Supplementary Figure S1).

For flupirtine:flupirtine-d_4_ ratios above 2:1, flupirtine concentrations in the calibration curve were fixed at 5 pmol on column (0.42 µg/mL flupirtine maleate) and the flupirtine-d_4_ concentrations were reduced. When possible, the same calibration curve was used to calculate the amount of D-13223 concentrations in the samples. Samples with trace concentrations of D-13223 were quantified using a separate external calibration curve ranging from 1 to 1000 fmol of D-13223 on column (0.063–62.5 ng/mL of D-13223 HCl). The MS/MS response for D-13223 was linear over the entire range. For the external standard curve of D-13223, the LLOQ was 0.5 fmol (accuracy = 129.4%, RSD for concentration estimation = 36.5%, 0.031 ng/mL of D-13223 HCl; N = 4). The area response for D-13223 at 0.5 fmol or 0.031 ng/mL had an RSD of 11.1%. 

Recovery of flupirtine after liquid–liquid extraction was ~80% in the brain tissues in the absence of correction with internal standard. Because we used flupirtine-d_4_ as an internal standard, any loss of analyte during extraction should result in an equal loss of flupirtine-d_4_, thereby correcting for loss on extraction and for variations in recovery from the tissues assessed. Due to the structural similarities between flupirtine and D-13223, recovery of D-13223 should be approximately the same as recovery of flupirtine, which allows flupirtine-d_4_ to also correct for the loss of D-13223.

As the flupirtine and D-13223 concentrations of right and left cortex, right and left hippocampus, left and right kidney, and left and right retina for in vivo distribution experiments were comparable, the results for left and right parts for each tissue type from the same animal were averaged and used as one sample. The concentrations of flupirtine as well as D-13223 in ex vivo samples were corrected for sample dilution at each sampling time, and averages were presented per milliliter of incubation medium. 

## 3. Data Analysis

The experimental results are represented as mean ± standard deviation. Statistical analysis was conducted using one-way ANOVA followed by Tukey’s post hoc analysis (GraphPad Prism, v 4.5, GraphPad Software, San Diego, CA, USA). The results were considered statistically significant at *p* < 0.05. 

Assuming first-order elimination, elimination rate constant and half-life were estimated for flupirtine. The area under the concentration vs. time curve (AUC_0–24h_) for flupirtine and D-13223 was estimated using a linear trapezoid rule.

## 4. Results

### 4.1. Biodistribution and Pharmacokinetics of Flupirtine 

The elimination rate constant and half-life of flupirtine in the tissues were calculated based on the flupirtine concentrations at 1-, 6-, and 24-h time-points (Table 1). The half-life of flupirtine ranged from 3.1 to 5.2 h in the various tissues studied. The AUC_0–24h_ ranked the highest in liver and the lowest in spleen for both flupirtine as well as D-13223 (Table 1).

**Figure 1:**
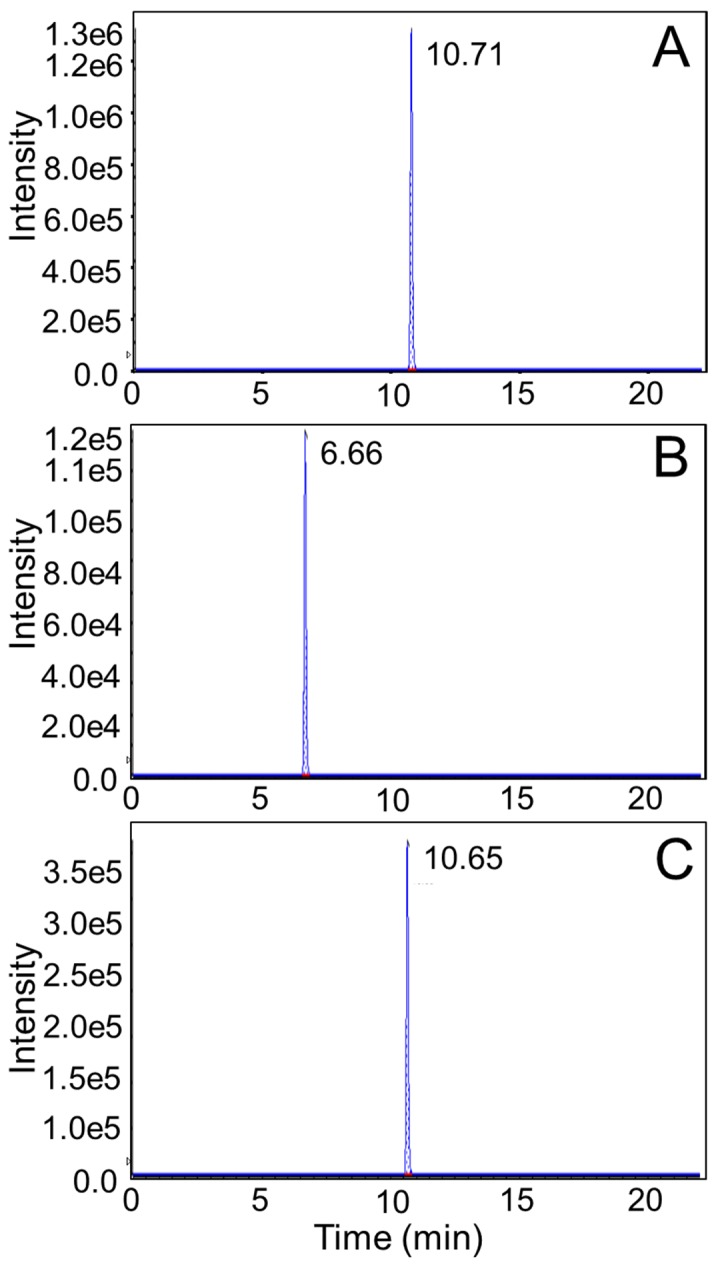
A representative LC-MS/MS chromatogram from a neonatal rat liver that was harvested 6 h after dosing with flupirtine. We measured 27.2 ng flupirtine/mg liver (**A**), 1.9 ng D-13223/mg liver (**B**), and 6.7 ng flupirtine-d_4_/mg liver (**C**) in this sample. The MS/MS transitions used were: *m/z* 305.1 → *m/z* 196.1 for flupirtine, *m/z* 275.2 → *m/z* 166.1 for D-13223, and *m/z* 309.1 → *m/z* 196.1 for flupirtine-d_4_.

**Figure 2:**
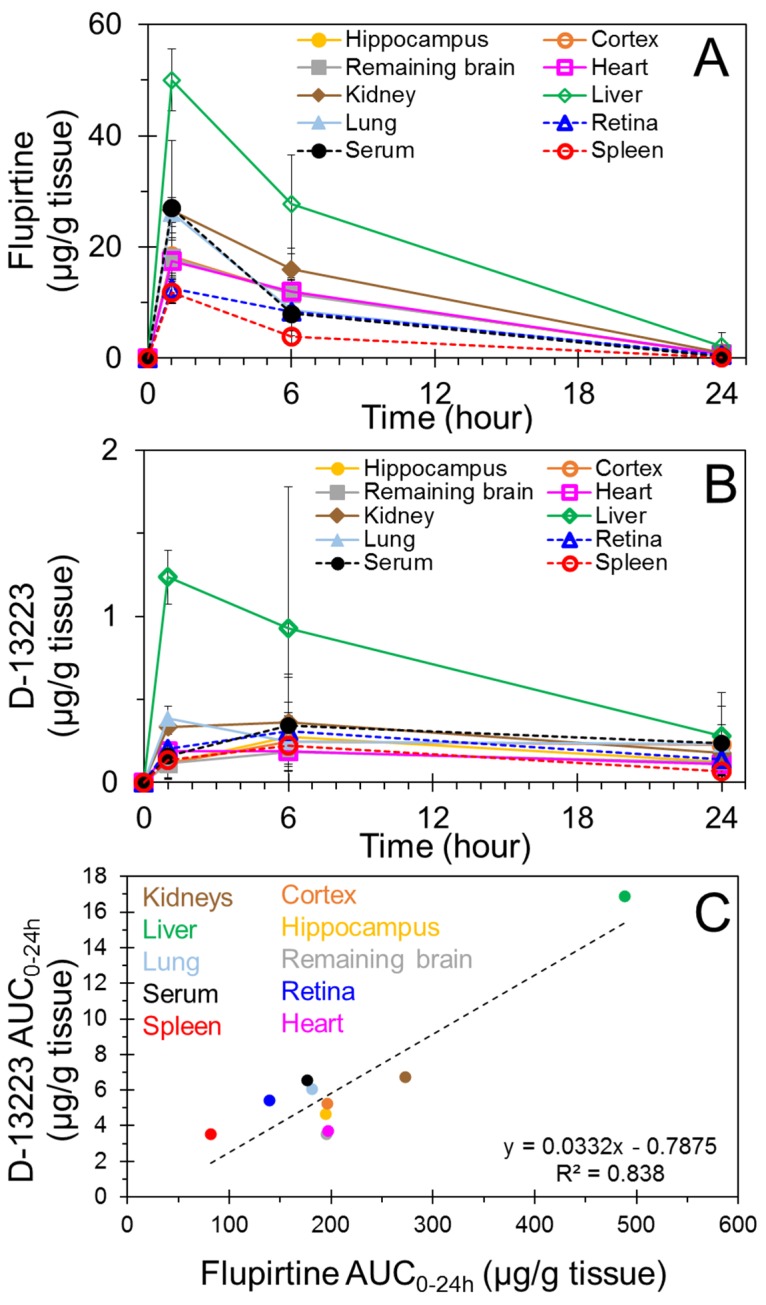
In vivo time-course of concentrations of flupirtine and its metabolite D-13223 in various tissues of neonatal rats and corresponding area under the concentration vs. time curve (AUC_0–24h_) following a systemic dose of 25 mg/kg flupirtine. (**A**) Flupirtine. (**B**) D-13223. (**C**) Correlation of AUC_0–24h_ for flupirtine and D-13224. Data is presented as mean ± SD for three to seven animals in panels A and B and as values based on average concentration-time-course in panel C.

Figure 1 shows a representative LC-MS/MS chromatogram for flupirtine, D-13223, and flupirtine-d_4_ (internal standard) from a neonatal rat liver harvested 6 h after dosing with flupirtine. Figure 2A shows flupirtine concentrations in neonatal rat tissues at 1, 6, and 24 h. Flupirtine was detectable at all time-points in each tissue type studied. Flupirtine concentrations in all the tissues were the highest at 1 h, followed by a gradual reduction at 6- and 24-h postdosing. At 1-h postdosing, liver had the highest concentration of flupirtine, while spleen had the lowest concentration. Liver concentrations of flupirtine at 1 and 6 h were significantly higher compared to all other tissues. Flupirtine concentrations were not significantly different between different brain regions at any time-point.

Figure 2B presents tissue concentrations of D-13223, a biologically active metabolite of flupirtine, at 1, 6, and 24 h after intraperitoneal administration of flupirtine. D-13223 could be quantified at all time-points in all the tissue types studied. Peak D-13223 concentrations occurred at 1 h for heart, liver, and lung tissues. Peak D-13223 concentrations occurred in all other tissues 6 h after dosing. The liver concentration of D-13223 was significantly higher than all other tissues at 1 h. At 1 h, the lung concentration of D-13223 was significantly higher than all brain regions. The kidney concentration of D-13223 at 1 h was significantly higher than the hippocampus and the remaining brain. D-13223 concentrations were not significantly different between different brain regions at any time-point.

AUC_0–24h_ of D-13223 correlated well with AUC_0–24h_ of flupirtine when all tissues were plotted, with a correlation coefficient (*R*^2^) of 0.84 (Figure 2C). Further, correlations between flupirtine and D-13223 concentrations at multiple time-points indicated *R*^2^ of 0.49 and 0.68 for liver and lung, respectively, with other tissues showing even lower correlation.

### 4.2. Conversion of Flupirtine to D-13223 in Isolated Rat Tissues 

**Figure 3:**
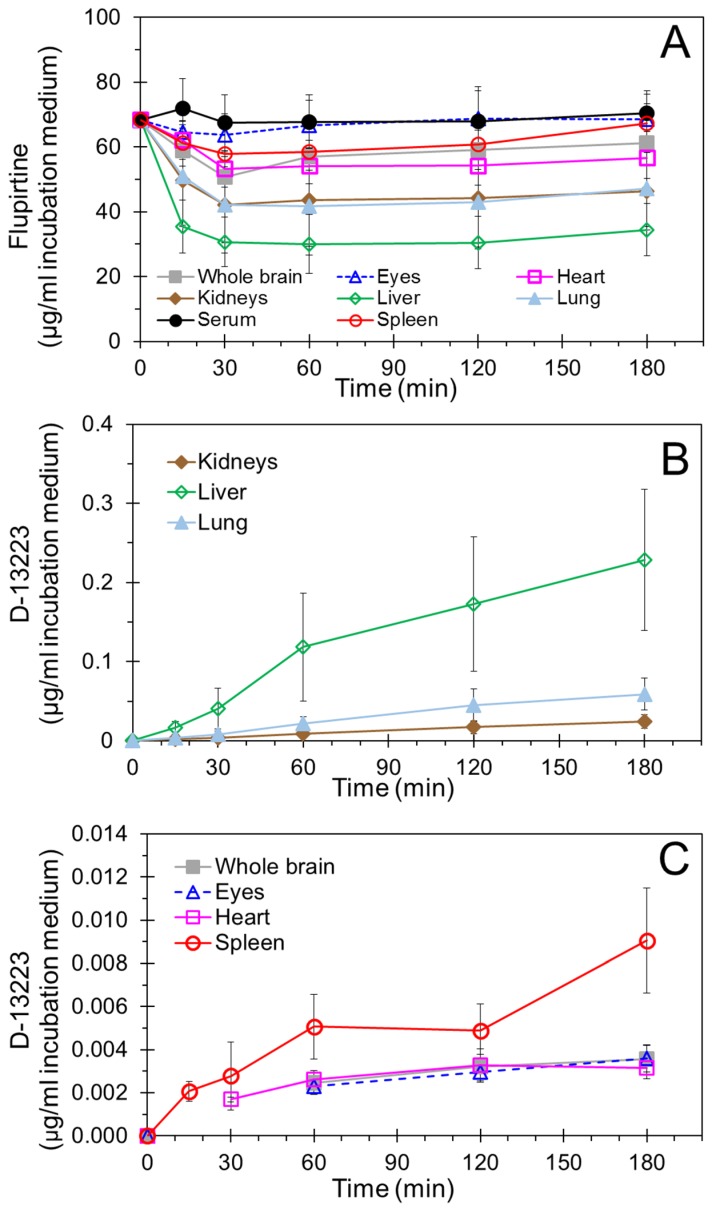
Ex vivo time-course of concentrations of (**A**) flupirtine and (**B** and **C**) metabolite D-13223 in the incubation medium of various tissues of neonatal rats. Isolated tissues were incubated with 60 µg of flupirtine in 1 mL PBS at 37 °C. Data is presented as mean ± SD for three to nine animals.

Figure 3 shows the time-course of flupirtine and D-13223 concentrations in the incubation medium. Flupirtine remained quantifiable in the incubation medium throughout the 3-h experiment for all the samples (Figure 3A). Flupirtine concentrations at 30 min were the highest for the serum and the lowest for the liver. After the initial rapid decline by 30 min, flupirtine concentrations remained relatively stable in the incubation medium for all the tissues. The drug loss was the highest in the incubation medium for the liver and the lowest for the serum, eyes, and spleen.

The amount of D-13223 present in the incubation medium at a given time was used as a measure of flupirtine metabolism (Figure 3B,C). D-13223 was detected in the incubation media of all tissues except serum, indicating that flupirtine was metabolized in all the isolated tissues except serum. All the tissue incubation media except that of serum showed gradual increase of D-13223 throughout the incubation period. The concentrations of D-13233 ranked the highest for the liver and the lowest for the heart and the whole brain at the 3-h time-point among the tissues that metabolized the drug. The liver medium concentration of D-13223 was significantly higher than all other tissues at each time-point. In Figure 3C, concentrations of D-13223 were below the limit of quantitation at 15- and 30-min time-points for some of the tissues in a group, reducing the number of tissues below three. These values were not included in the plot. The zero values represent the theoretical value at time zero.

### 4.3. Tissue Partitioning In Ex Vivo Vs In Vivo Studies

**Figure 4:**
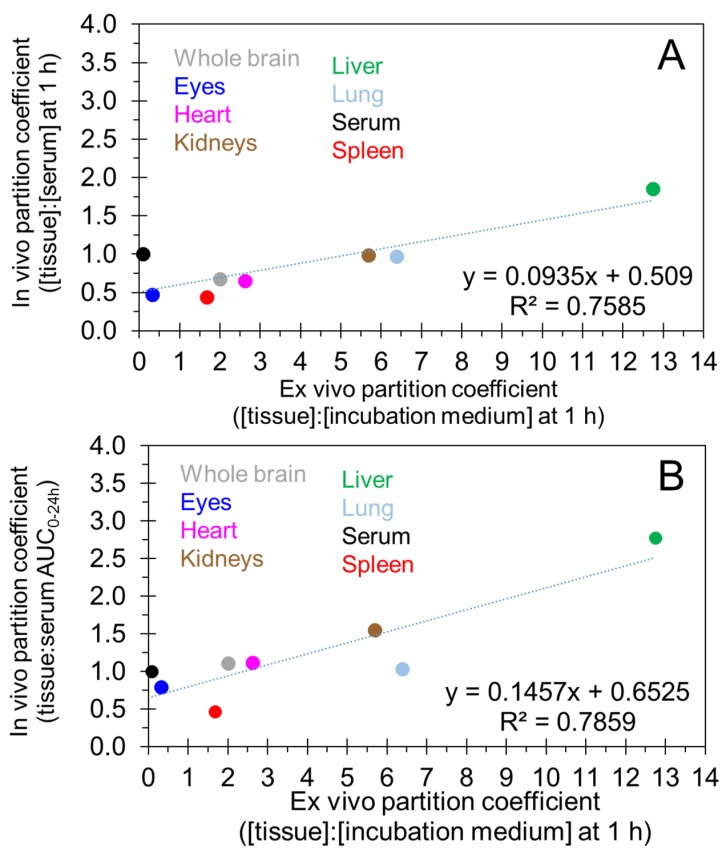
Correlation of in vivo and ex vivo partition coefficients of flupirtine obtained in various tissues of neonatal rats. (**A**) In vivo partition coefficients based on drug concentrations at 1 h vs. ex vivo partition coefficients. (**B**) In vivo partition coefficients based on AUC_0–24h_ vs. ex vivo partition coefficients. Ex vivo partition coefficients were based on drug concentrations at 1 h.

Figure 4 correlates tissue partition coefficient values measured ex vivo as tissue:medium drug concentration ratio at 1 h with in vivo partition coefficients measured as tissue:serum ratio of drug concentrations at 1 h (Figure 4A) or AUC_0–24h_ (Figure 4B). Although the magnitudes of ex vivo and in vivo partition coefficients were different, with the ex vivo values being higher, the correlations were good with *R*^2^ of 0.76 and 0.79 for in vivo tissue concentration and AUC-based measures, respectively.

## 5. Discussion

**Figure 5:**
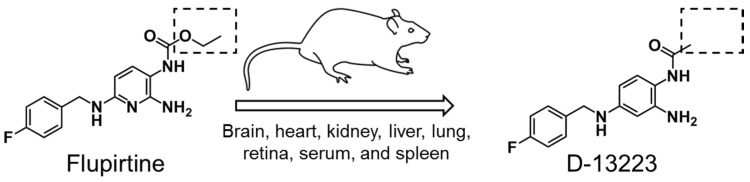
Bioconversion of flupirtine to D-13223 in vivo in neonatal rats. The rectangle regions marked by dotted lines represent the difference between the two structures.

Although flupirtine mediates its analgesic activity mainly via its action at the central nervous system level, no publication has reported flupirtine concentrations and its half-life in the brain. This is the first study to directly quantify flupirtine concentrations in the brain tissue and determine flupirtine pharmacokinetics in neonate animals. We measured flupirtine and its active metabolite D-13223 in multiple tissues using a highly sensitive LC-MS/MS method, following intraperitoneal administration of flupirtine in neonatal rats. Recent studies in animal models suggest that flupirtine is very effective against hypoxia–ischemia-induced neonatal seizures [15,16]. In newborns, seizures are most commonly associated with a hypoxic–ischemic event that often injures multiple brain regions such as the hippocampus and cortex [25]. These regions are potential targets for flupirtine in treating hypoxic–ischemic encephalopathy. Our results suggest that flupirtine was delivered to all brain regions assessed, including the hippocampus and cortex. Furthermore, flupirtine was distributed to nonbrain tissues including the liver, kidney, lung, heart, spleen, and retina, with the half-life being a few hours in all tissues assessed (Figure 2A, Table 1). Flupirtine formed its biologically active metabolite D-13223 in vivo in neonatal rats, with the metabolite detectable in all tissues analyzed (Figure 2B and Figure 5). Isolated tissue studies indicated drug-to-metabolite conversion in all tissues assessed, except serum (Figure 3). 

While the intravenous route is the most common route for administering drugs to treat neonatal seizures in the clinic, tail vein injection is technically very challenging and unreliable in neonatal rat pups due to their small size. Therefore, we selected the intraperitoneal route of administration for the current study. For determining the approximate dose of flupirtine, the US Food and Drug Administration (FDA) guidance on interspecies scaling factor was used. The approved oral dose of flupirtine for analgesia in humans is 100–400 mg/subject/day [26,27]. Considering the average human daily dose of flupirtine is 250 mg, which approximately equals 4 mg/kg, and based on scaling factor 6.2 for rats [28], the daily dose for an adult rat was estimated to be about 25 mg/kg. Therefore, the 25 mg/kg dose used in the current study was the human equivalent dose for adult rats. At this dose, no gross side effects or behavioral changes were observed in animals during the experimental period. Future studies will assess dose dependency of drug and metabolite pharmacokinetics. 

With the intraperitoneal mode of administration used in this study, the drug was absorbed into the liver via portal circulation at first, followed by entry into the systemic circulation. Consistent with this first-pass effect, liver accumulation of the drug was the highest, followed by other tissues. Given the high oral bioavailability of flupirtine [17], it is likely that the drug was well absorbed from the intraperitoneal route. Drug concentrations were the highest at 1 h in all tissues, suggesting rapid absorption of the drug. Beyond 1 h, the drug concentrations declined, possibly because drug elimination outweighed drug absorption (Figure 2A). 

While octanol:water partition coefficients are typically measured and reported for most new drug molecules, reports on measured tissue partition coefficients are less common. In this study, we estimated the partition coefficient for flupirtine in various tissues using ex vivo and in vivo approaches (Figure 4A,B). For the ex vivo approach, a ratio of drug concentration between a tissue and the incubation medium was used. For the in vivo approach, tissue:serum drug concentration ratios at 1 h or AUC_0–24h_ ratios were estimated as measures of partition coefficient. Ex vivo measures were higher for all tissues but correlated well with the two in vivo measures of partition coefficient. The results indicated the highest partitioning of the drug into the liver based on all the above approaches. The brain also exhibited a high partition coefficient. 

Flupirtine is known to be metabolized by liver enzymes, including esterases and *N*-acetyl transferase to D-13223, which has about a fourth of the analgesic activity as flupirtine [29]. While esterases are ubiquitous in various tissues in the body, *N*-acetyl transferase, an enzyme capable of converting a flupirtine intermediate to D-13223 [21], has been detected in the liver, lung, kidney, heart, and spleen of rats [30]. We measured D-13223 formation in vivo as well as ex vivo (Figure 2 and Figure 3). These studies confirmed that all tissues except serum metabolize flupirtine to D-13223, with the greatest extent of metabolism taking place in the liver. With the small number of male and female animals used in this study, no significant differences were evident between the sexes for flupirtine or D-13223 concentrations in vivo or ex vivo. 

The half-life of flupirtine ranged from 3.1 to 5.2 h in all the tissues analyzed (Table 1). Prior studies reported that the half-life of flupirtine is 8.5–10.7 h in plasma after single intravenous, oral, and rectal administrations in humans [17] and 2.2 h in adult rat and 2.6 h in dog after oral administration [21]. Since liver concentrations of the metabolite were the highest and showed a continuous decline, we estimated the half-life of D-13223 using all the time-points where the metabolite concentrations were measured. The estimated half-life of D-13223 from liver tissues was 10.7 h, which may represent the rate of metabolism to D-13223 rather than its elimination.

A limitation of the current study was the small number of time-points used for the determination of the half-life of flupirtine and D-13223. Additionally, the use of the intraperitoneal route in the current study as opposed to an intravenous route may affect half-life estimates. Since tail vein injections in neonatal rats are technically challenging and not reliable, they were not used in this study. A limitation of flupirtine is that it induces acute liver injury in 0.01% of patients, and about seven deaths have been reported following dosing of >17 million average daily prescriptions [31,32]. Furthermore, for most patients, the onset of liver injury is delayed, with the changes in liver function observed after four months of dosing [31]. Thus, it is unlikely that the liver function was affected by flupirtine within 24 h, which was the duration of the present study. 

The present study is the first study to assess flupirtine pharmacokinetics in various tissues other than whole blood/plasma. Prior studies assessed the pharmacokinetics of flupirtine based on its plasma concentrations [18]. Furthermore, to the best of our knowledge, there are no earlier published references showing flupirtine tissue distribution. Our study is among the very few studies that assessed the pharmacokinetics of any drug in neonatal animals [33,34,35]. Our findings suggest that flupirtine rapidly enters the brain and other tissues and is metabolized by the brain as well as other tissues, with the liver being the primary organ responsible for metabolism. Drug distribution is similar between the cortex, hippocampus, and the remaining brain, suggesting no additional barriers for drug entry into target tissues once the drug is past the blood brain barrier. Drug concentrations persisted for at least 24 h in all tissues, including the brain. However, most of the drug was eliminated within the first few hours. Previous studies by Dr. Raol’s group indicated that a single intraperitoneal dose of flupirtine is capable of reducing neonatal seizures in a hypoxia–ischemia rat model [16]. However, they found that a single dose was not sufficient and daily dosing over a few days was required to prevent clinical seizures that occur over a 72-h period following hypoxia–ischemia induction [16]. This study, while supportive of daily dosing based on the presence of drug for at least 24 h in the brain, also suggests that a more frequent dosing or sustained delivery might be more beneficial. Future studies should assess the benefit of slow release delivery systems or more frequent dosing to treat neonatal seizures using flupirtine. 

## Figures and Tables

**Table 1:** Pharmacokinetic parameters of flupirtine in neonatal Sprague-Dawley rats following systemic dosing at a dose of 25 mg/kg. Pharmacokinetic parameters were calculated using mean concentration–time-course data (*n* = 3–7). NE—Not estimated.

Tissue	Flupirtine			D-13223	
	AUC_0–24h_ (µg·h/g Tissue)	Elimination Rate Constant (k_e_, h^−1^)	Half-Life (t_1/2_, h)	AUC_0–24h_ (µg·h/g Tissue)	Half-Life (t_1/2_, h)
Hippocampus	195	0.140	4.9	4.70	NE
Cortex	196	0.142	4.9	5.25	NE
Remaining brain	195	0.135	5.1	3.56	NE
Heart	197	0.145	4.8	3.75	NE
Kidney	273	0.144	4.8	6.74	NE
Liver	488	0.137	5.1	16.9	10.7
Lung	181	0.179	3.9	6.09	NE
Retina	139	0.134	5.2	5.43	NE
Serum	176	0.192	3.6	6.56	NE
Spleen	82	0.222	3.1	3.54	NE

## Supplementary Materials

The following are available online at http://www.mdpi.com/1999-4923/10/4/281/s1, Figure S1. (A) An LC-MS/MS analysis of control liver extract in MRM mode spiked with flupirtine-d_4_ (blue; 125 fmol on column) as a retention time marker. No interfering peaks were detected. (B) The analysis was repeated with a postcolumn infusion (10 mL/min) of a 25 nM (10.51 ng/mL) of flupirtine maleate solution and 25 nM (7.77 ng/mL) of D-13223 HCl solution mixed into the column eluent through a three-way junction. The signal intensity for flupirtine (green) and D-13223 (red) slowly tracks with the gradient. No matrix-based signal enhancement or suppression was detected.
