# Supplementary material for: Brain Distribution and Metabolism of Flupirtine, a Nonopioid Analgesic Drug with Antiseizure Effects, in Neonatal Rats
Source: Pharmaceutics. 2018 Dec 16;10(4):281. doi: 10.3390/pharmaceutics10040281 (PMC6320943; doi:10.3390/pharmaceutics10040281)
Supplement: Supplementary file 1 [file pharmaceutics-10-00281-s001.pdf]

pharmaceutics

MDPI

Brain Distribution and Metabolism of Flupirtine, a Nonopioid Analgesic Drug with Antiseizure Effects, in Neonatal Rats

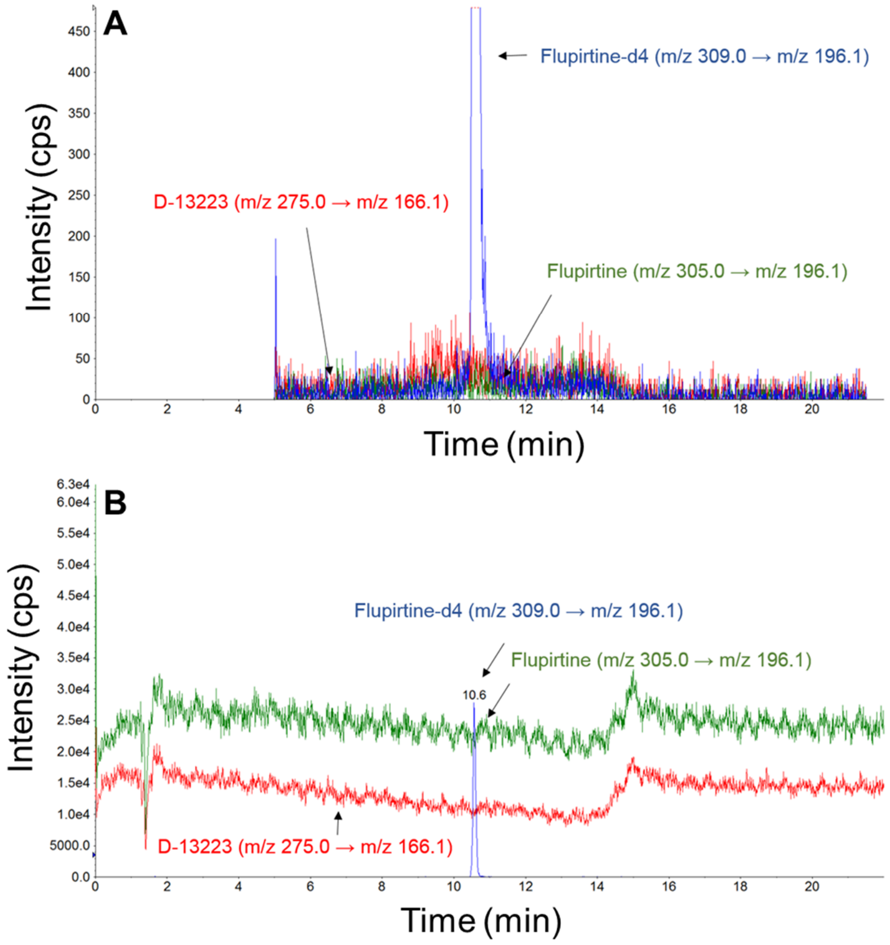

Figure S1. (**A**) An LC-MS/MS analysis of control liver extract in MRM mode spiked with flupirtine-d_4 (blue; 125 fmol on column) as a retention time marker. No interfering peaks were detected. (**B**) The analysis was repeated with a postcolumn infusion (10 mL/min) of a 25 nM (10.51 ng/mL) of flupirtine maleate solution and 25 nM (7.77 ng/mL) of D-13223 HCl solution mixed into the column eluent through a three-way junction. The signal intensity for flupirtine (green) and D-13223 (red) slowly tracks with the gradient. No matrix-based signal enhancement or suppression was detected.
